# Inheritance of DNA Methylation Patterns and Its Role in Modulating Allelic Expression in *Camellia* F_1_ Hybrids

**DOI:** 10.3390/plants15010094

**Published:** 2025-12-28

**Authors:** Min Zhang, Lin-Jian Xie, Shu-Rong Yan, Qi-Ling Huang, Cai-Lin Xu, Zi-Fei Li, Yi-Wei Tang, Xin-Kai Liu, Nai-Sheng Zhong, Wen-Ju Zhang

**Affiliations:** 1 Co-Innovation Center for Sustainable Forestry in Southern China, Nanjing Forestry University, Nanjing 210037, China; 8221711759@njfu.edu.cn (L.-J.X.); 3231700717@njfu.edu.cn (S.-R.Y.); qilinghuang70@163.com (Q.-L.H.); cailinxu001@163.com (C.-L.X.); 8251812008@njfu.edu.cn (Z.-F.L.); 2 College of Life Sciences, Nanjing Forestry University, Nanjing 210037, China; 3 Ministry of Education Key Laboratory for Biodiversity Science and Ecological Engineering, Institute of Biodiversity Science, School of Life Sciences, Fudan University, Shanghai 200438, China; 16210700094@fudan.edu.cn (Y.-W.T.); wjzhang@fudan.edu.cn (W.-J.Z.); 4 Palm Eco-Town Development Co., Ltd., Guangzhou 510627, China; lxk1000@163.com (X.-K.L.); zhongnsh@163.com (N.-S.Z.)

**Keywords:** allelic expression imbalance, hybridization, SNP, WGBS

## Abstract

DNA methylation, as an important epigenetic modification, plays a key role in shaping hybrid phenotypes. Studies have shown that DNA methylation—specifically, allele-specific methylation (ASM)—can mediate allelic expression imbalance (AEI) and participate in the regulation of plant growth and development. However, since this regulatory mechanism is often sequence-dependent, the prevalence of ASM and the extent to which it influences allelic expression remain poorly characterized. To address this challenge, the present study utilized *Camellia azalea*, *C. amplexicaulis* and their F_1_ hybrids [*C. azalea* (♀) × *C. amplexicaulis* (♂)] as research materials. By performing whole-genome bisulfite sequencing (WGBS), resequencing, and transcriptome sequencing, we assessed the inheritance of DNA methylation patterns and its role in shaping allelic expression in F_1_ hybrids. The results showed the following: (1) the overall cytosine methylation level in the F_1_ hybrid was intermediate between the two parents; (2) the methylation states of the parental genomes were partly transmitted to the next generation; (3) ASM was not prevalent in the F_1_ hybrids, primarily because biparental parent-specific methylation sites (PSMSs) were widespread and randomly distributed, which often act on the same allele pairs; (4) although ASM was not common, it led to biased expression of some alleles related to flower development. The results indicated that ASM was rare in F_1_ hybrids, mainly because PSMSs occurred randomly. Instead of causing AEI, the randomly distributed PSMSs played a more important role in balancing allelic expression in F_1_ hybrids. Therefore, most of the alleles in F_1_ were not biasedly expressed. ASM did not necessarily lead to allele-biased expression; however, its occurrence may hold significant biological implications in modulating AEI and transgressive phenotypes in the F_1_ hybrids. These findings elucidate the synergistic effects of genetic and epigenetic controls on transcriptional regulation in hybrid plants, substantially deepening the mechanistic understanding of hybridization at the molecular scale.

## 1. Introduction

Hybridization refers to the process of crossing different species or varieties to produce hybrids. In nature, hybridization is prevalent. Recent genomic studies indicate that 8% to 25% of extant angiosperms show evidence of interspecific hybridization or ongoing introgression [1]. Compared to their homoploid parental lineages, hybrids generated from hybridization often exhibit transgressive phenotypes, such as enhanced growth, yield, and environmental adaptability, which may significantly drive ecological adaptation and speciation [2,3,4]. In breeding practices, hybridization is one of the most widely applied strategies for crop improvement, enabling the rapid generation of numerous genetically diverse varieties within a short period [5]. Nevertheless, the underlying mechanism for the origin of novel traits during hybridization remains obscure. Understanding the molecular mechanisms driving hybridization is key to elucidating how phenotypic diversity arises in hybrids and ultimately contributes to speciation. This continues to be a research hotspot in both botanical and breeding sciences.

Gene expression divergence is a fundamental driver of phenotypic novelty in hybrids. Recent advances in genomics have revealed that the merging of parental genomes during hybridization can trigger “transcriptomic shock”, characterized by two diagnostic features: (1) non-additive expression, where the gene expression of hybrids deviates from mid-parent values, and (2) allelic expression imbalance (AEI), referring to asymmetric expression of homologous alleles [6,7]. Notably, mounting evidence underscores AEI’s central role in generating novel hybrid phenotypes: in rice (*Oryza sativa*), AEI quantitatively correlates with heterosis magnitude [8]; in pepper (*Capsicum*), it directly drives fruit morphological divergence between cultivated and wild varieties [9]; in maize (*Zea mays*), it determines grain yield traits [10]. AEI theory enriches and expands the connotation of classical genetic laws, providing new insights into understanding the formation mechanisms of unique hybrid phenotypes.

**Figure 1:**
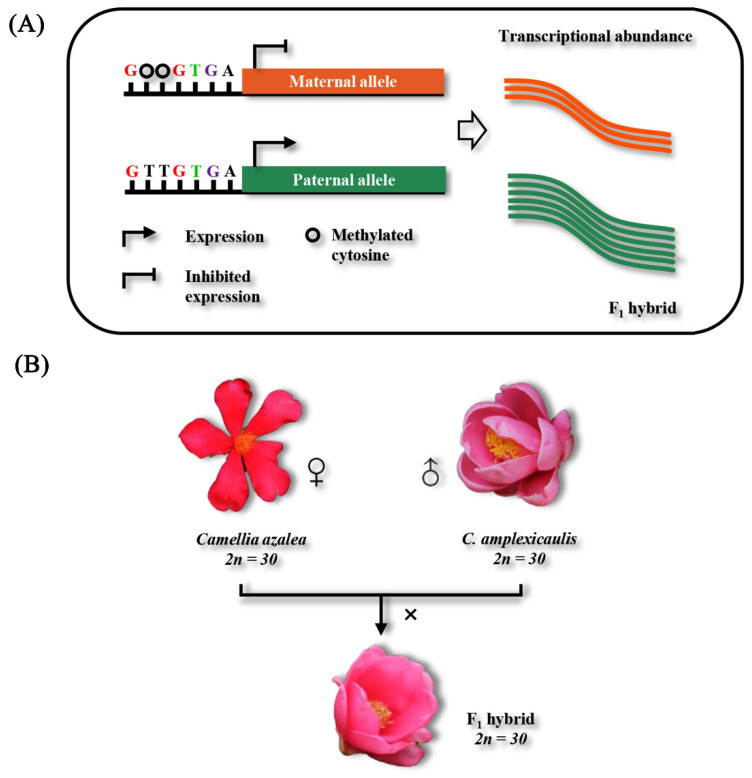
Scientific hypothesis and research materials of this study. (**A**) A basic model demonstrating allelic expression imbalance induced by allele-specific DNA methylation. In the figure, the maternal allele harbors species-specific cytosine sites; methylation at these sites represses its expression, ultimately causing allelic expression imbalance. Modified from Cleary and Seoighe [7] (**B**) Diagram showing the construction of the *Camellia* hybrid as well as materials used in this study.

AEI is often driven by a range of genetic and epigenetic factors [11], of which DNA methylation is an epigenetic marker that plays a direct role in transcriptional regulation. Beyond its genome-wide impact on transcriptional profiles, DNA methylation can also exhibit allele-specific patterns that are mechanistically associated with AEI [7,12]. This occurs because the methylation status of different alleles is likely controlled in *cis* by the local DNA sequence [12,13]. It is foreseeable that alleles with species-specific cytosines exhibit higher susceptibility to methylation, rendering their expression more susceptible to methylation-mediated suppression and ultimately leading to AEI (Figure 1A). Studies on herbaceous crops such as rice (*O*. *sativa*) [14] and maize (*Z*. *mays*) [15] have provided preliminary support for this model. By analyzing DNA methylomes and transcriptomes of the hybrid rice and its parental varieties, Ma et al. [14] found that DNA methylation is closely linked with AEI. Similarly, intraspecific whole-genome correlation analysis between DNA methylation and imprinted genes indicated that DNA methylation plays an important role in imprinting variants, an extreme case of AEI [16]. However, herbaceous plants have short life cycles, whereas woody plants have long lifespans. DNA methylation modifications induced by hybridization may persist throughout woody plants’ prolonged life history, leading to patterns distinct from herbaceous plants. Thus, evidence from woody plants is needed to clarify how DNA methylation regulates allelic expression. Additionally, previous studies mainly focused on allele-specific methylation (ASM) of a limited number of specific genes. Since methylation establishment is genetically dependent and interacts with genetic factors [12,13], while genetic variation is often random, the genome-wide extent and universality of DNA methylation’s impact on allelic expression remain unclear. Therefore, research quantifying this regulatory role is crucial for understanding how genetic and epigenetic factors synergistically regulate allelic expression.

Tackling this issue, two woody species of *Camellia azalea* C. F. Wei, *C. amplexicaulis* (Pitard) Coh. Stuart. as well as their F_1_ offsprings [*C. azalea* (♀) × *C. amplexicaulis* (♂)] (Figure 1B) were used to study the cytosine methylation dynamics and associated epigenetic effects in synthetic *Camellia* hybrids. Through utilizing an integrated multi-omics approach encompassing whole-genome bisulfite sequencing (WGBS), resequencing, and transcriptome profiling, we aimed to elucidate the inheritance patterns of cytosine methylation during interspecific hybridization and quantitatively assess their functional consequences on AEI. Our findings elucidate the synergistic effects of genetic and epigenetic controls on transcriptional regulation in hybrid plants, substantially deepening the mechanistic understanding of hybridization at the molecular scale.

## 2. Results

### 2.1. Morphological Divergence Between the F_1_ Hybrids and Their Parents

In this study, two species of *C. azalea*, *C. amplexicaulis* as well as their F_1_ hybrids [*C. azalea* (♀) × *C. amplexicaulis* (♂)] were used to study the epigenetic change after hybridization. As shown in Figure 1B, the F_1_ hybrids exhibited significant morphological divergence from their parental lines. According to our phenotypic research [17], most morphological indicators, including petals per flower, petal length, and stamens per flower in the F_1_ hybrids, displayed intermediate values between the parents. However, petal width and style length were enhanced, while sepals per flower were reduced in the F_1_ hybrids. Thus, the F_1_ generation exhibits both homology and differentiation in floral traits compared to their parental lines.

### 2.2. Whole-Genome Bisulfite Sequencing and Data Alignment

Whole-genome bisulfite sequencing (WGBS) was applied to detect the global cytosine methylation divergence induced by hybridization. Three sequencing libraries were constructed, generating 321.51 Gb clean data, with an average of 107.17 Gb per sample (Table 1). The clean reads were then aligned to the reference genome of *C. sinensis* [18]. The mapping rates for the male parent, female parent lines and F_1_ hybrids were 74.46%, 74.18% and 75.44%; the uniquely mapped rates were 54.71%, 52.94% and 54.37%, respectively. As shown in Table 1, the sequencing achieved an average depth of 16.57~17.12-fold coverage across the genome. These results indicated that the species involved in this study are closely related to the reference genome, and the sequencing depth is sufficient to ensure the accuracy of downstream analyses.

### 2.3. The Global Methylation Profiles of the Parental Lines and F_1_ Hybrids

**Figure 2:**
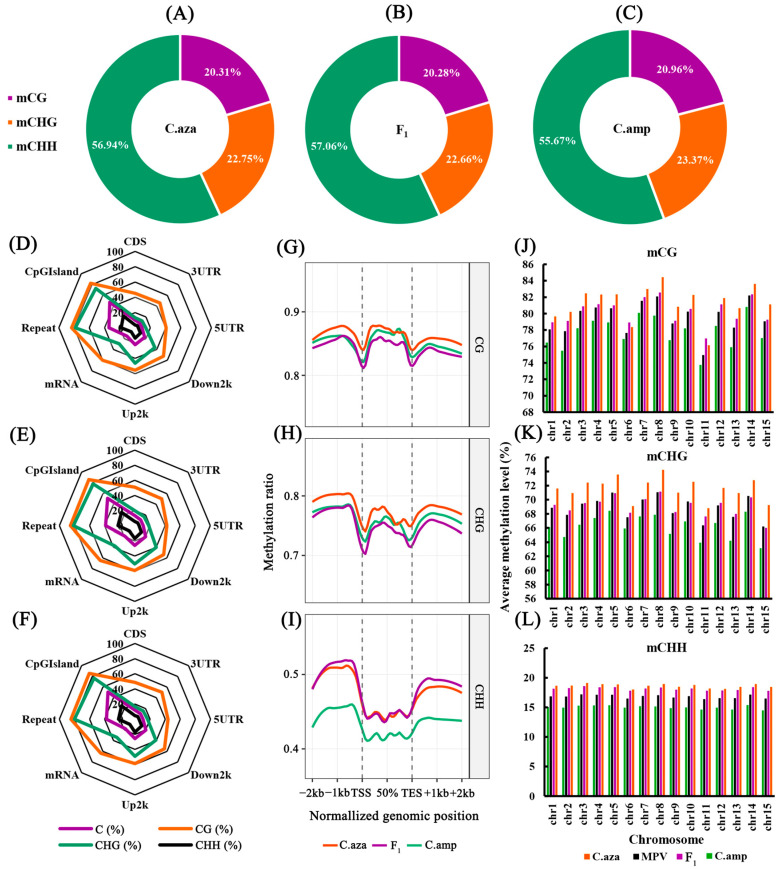
The global methylation profiles of the parental lines and F_1_ hybrids. (**A**–**C**) Proportion of methylated cytosines, including mCG, mCHG and mCHH, in *Camellia azalea* (**A**), the F_1_ hybrids (**B**), and *C. amplexicaulis* (**C**). (**D**–**F**) Average methylation level across different elements of *C. amplexicaulis* (**D**), *C. azalea* (**E**), and the F_1_ hybrids (**F**) in CG, CHG, and CHH contexts. (**G**–**I**) Metaplot showing regional DNA methylation ratio of genes in CG (**G**), CHG (**H**), and CHH (**I**) contexts. TSS, transcription start sites; TES, transcription end sites. (**J**–**L**) Column diagrams showing the average methylation levels across chromosomes in CG (**J**), CHG (**K**), and CHH (**L**) contexts. C.aza, *C. azalea*; C.amp, *C. amplexicaulis*; F_1_, F_1_ hybrids; MPV, mid-parent value.

Whole-genome bisulfite sequencing identified 209.99 million, 167.17 million and 216.31 million methylated cytosines (mCs) from the male parent, female parent and F_1_ hybrid, respectively (Table 2). Among these, 20.28~20.96% were located in the CG context, while 22.66~23.37% and 55.68~57.06% in the CHG and CHH contexts (Figure 2A–C). Comparative analysis of methylation patterns across genomic contexts showed that CG, CHG, and CHH sites in all three samples exhibited maximal methylation enrichment in CpG islands and repetitive genomic regions. Specifically, CG sites displayed hypermethylation (>80%) in these regions, and CHG sites maintained consistently high methylation levels (>70%) (Figure 2D–F). In contrast, CHH methylation levels were markedly lower (18.23~24.85%) than those of CG and CHG contexts. Moreover, methylation patterns exhibited significant heterogeneity across distinct gene transcriptional elements (Figure 2G–I). Notably, upstream and downstream regions displayed higher methylation levels compared to the CDS (coding sequence) and UTR regions, regardless of cytosine context, and the patterns were roughly similar in all examined samples. Chromosome-wide analysis revealed that the F_1_ hybrids’ average methylation levels were intermediate between the two parental lines across most chromosomes. However, methylation levels of nearly all chromosomes in hybrids were higher than the mid-parent values (MPVs) in CG, CHG, and CHH contexts (Figure 2J–L).

### 2.4. Differentially Methylated Regions Between the Parental Lines and F_1_ Hybrids

**Figure 3:**
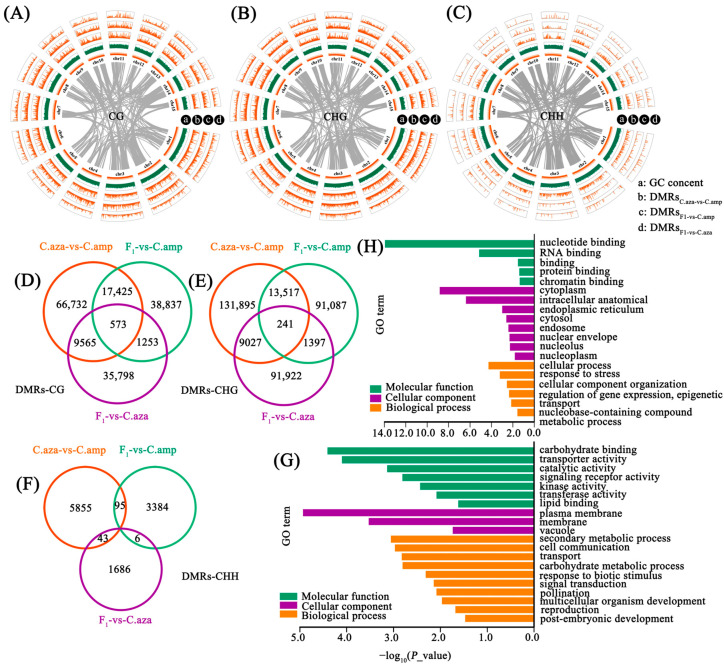
Differentially methylated regions between the parental lines and F_1_ hybrids. (**A**–**C**) Distribution of pairwise differentially methylated regions (DMRs) across the genome in CG (**A**), CHG (**B**), and CHH (**C**) contexts. (**D**–**F**) Venn diagram showing the number of DMRs shared by the pairwise comparison in CG (**D**), CHG (**E**), and CHH (**F**) contexts. (**G**) GO terms enriched from genes related to DMRs between F_1_ hybrids and the female parent in CG context. (**H**) GO terms enriched from genes related to DMRs between F_1_ hybrids and the male parent in CHG context. C.aza, *Camellia azalea*; C.amp, *C. amplexicaulis*; F_1_, F_1_ hybrids.

A sliding-window approach was employed to detect differentially methylated regions (DMRs). Figure 3A–C illustrate the DMRs identified through pairwise comparisons between F_1_ hybrids and their parents. Between the parents of *C. azalea* and *C. amplexicaulis*, we detected 94,295 CG, 154,680 CHG, and 5993 CHH DMRs. Comparisons between F_1_ hybrids and their male parent revealed 58,088 CG, 106,242 CHG, and 3485 CHH DMRs, while hybrids versus the female parent showed 47,189 CG, 102,587 CHG, and 1735 CHH DMRs. In the CG context, 10.14% (9565/94,295) of the parental CG DMRs were stably transmitted to the F_1_ hybrids and exhibited a methylation pattern similar to that of the paternal parent, whereas a higher proportion (18.48%, 17,425) were similar to that of the maternal parent (Figure 3D). The CHG context displayed a similar inheritance pattern (Figure 3E), with 8.74% (13,517/154,680) of parental CHG DMRs in F_1_ hybrids matching the mother’s methylation pattern, compared to 5.84% (9027) matching the father’s methylation pattern. In contrast, the proportion of paternal DMRs transmitted to the offspring showed a significant decrease at CHH sites compared to those in the CG and CHG contexts (Figure 3F). In addition, numerous differentially methylated regions emerged between the parents and offspring, which were not present in the parental generation (Figure 3D–F). DMRs are likely to play a functionally important role in shaping hybrid offspring phenotypes. To identify DMR-associated genes and their functional implications, we performed Gene Ontology (GO) enrichment analysis. The results revealed that DMR-related genes were significantly enriched in multiple biological processes. In the CG context, DMRs between F_1_ hybrids and the female parent were found to influence genes associated with “multicellular organism development,” “reproduction,” and “post-embryonic development” (Figure 3G). In the CHG context, DMRs between F_1_ hybrids and the male parent were linked to genes involved in “regulation of gene expression (epigenetic)”, “metabolic processes”, and other key biological pathways (Figure 3H).

### 2.5. Differentially Methylated Sites Between the Parental Lines and F_1_ Hybrids

**Figure 4:**
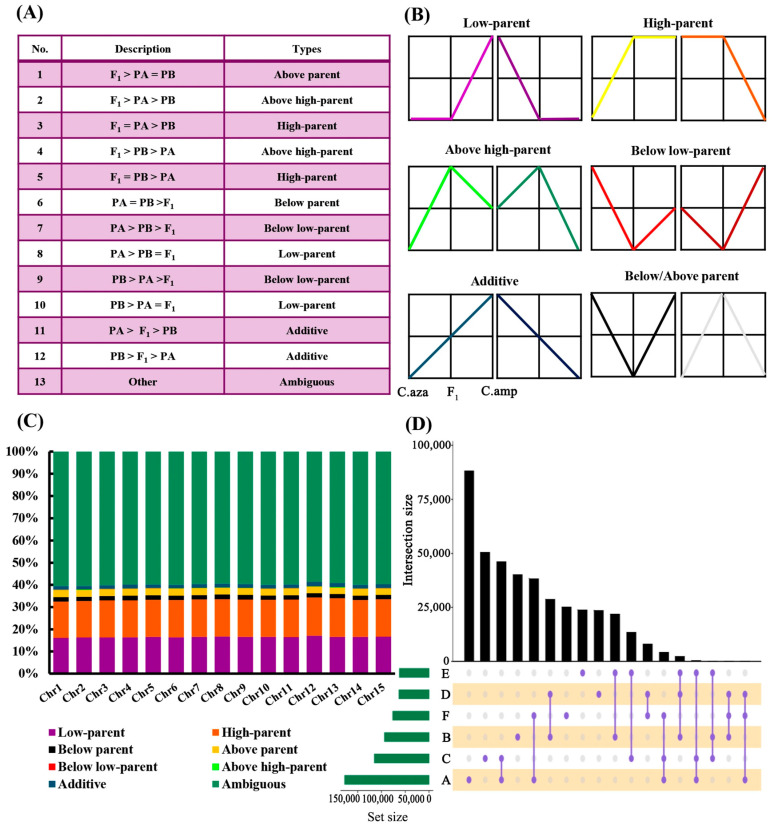
Classification and proportion of differentially methylated sites (DMSs) between F_1_ hybrids and parental lines. (**A**) Classification of differentially methylated sites between F_1_ hybrids and their parents. PA and PB represent two parents; F_1_, F_1_ hybrids. (**B**) Line plot showing distinct DNA methylation inheritance patterns after hybridization. (**C**) Stacked column chart showing the proportion of different DMS types across all chromosomes. (**D**) Upset plot showing the number of different DMSs in chromosome 1, where A to F represents DMSs with methylation level C.amp < C.aza, C.amp > C.aza, C.amp < F_1_, C.amp > F_1_, C.aza < F_1_, C.aza > F_1_, respectively. C.aza, *Camellia azalea*; C.amp, *C. amplexicaulis*; F_1_, F_1_ hybrids.

To elucidate the comprehensive inheritance patterns of cytosine methylation during hybridization, we performed Fisher’s exact test to identify pairwise differentially methylated sites (DMSs) between F_1_ hybrids and their parental lines. The pairwise DMSs were categorized into eight groups (Figure 4A,B), including “Additive”, “Low-parent”, “High-parent”, “Above high-parent”, “Below low-parent”, “Above parent”, “Below parent”, and “Ambiguous” (see Section 4.5 for definitions). As shown in Figure 4C, the proportions of different DMS types were largely consistent across all chromosomes. Specifically, discard DMSs could not be clearly defined algorithmically (59.45–60.52%); “High-parent” (16.35–17.39%) and “Low-parent” (16.17–17.09%) predominated among the non-additive DMSs. The remaining categories included “Above parent” (2.91–3.25%), “Below parent” (1.90–2.13%), “Above high-parent” (0.08–0.13%) and “Below low-parent” (0.05–0.08%). Additive DMSs accounted for 1.54–2.00% of the total DMSs. Take chromosome 1 (Chr1) as an example (Figure 4D); after excluding undefined DMSs (59.78%), 16.78% and 16.55% of the DMSs matched the “High-parent” and “Low-parent”, respectively. These were followed by 3.08% (“Above-parent”), 1.99% (“Below-parent”), 0.06% (“Below low-parent”), and 0.11% (“Above high-parent”). Additively inherited DMSs accounted for only 1.65%.

### 2.6. Parent-Specific Site Identification and Allelic Expression Analysis

**Figure 5:**
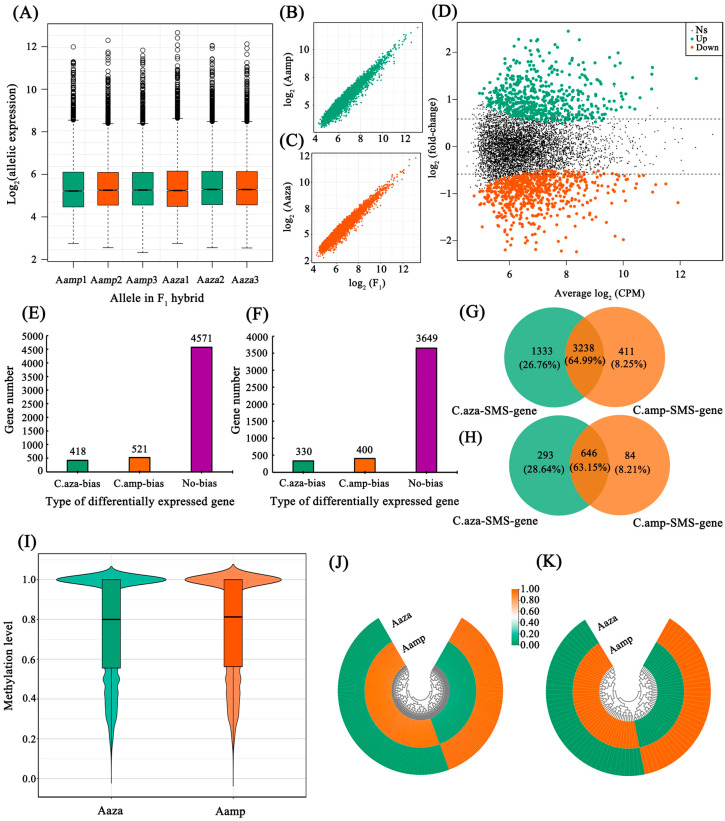
Diagrams showing the relationship between parent-specific methylation sites (PSMSs) and allelic expression imbalance (AEI). (**A**) Box plot showing the allelic expression after normalization. (**B**,**C**) Correlation between paternal (**B**) and maternal (**C**) monoallelic expression and F_1_ overall expression levels. (**D**) Volcano plot showing the divergent expression between the maternal and paternal alleles in F_1_ hybrids. NS, not significant. (**E**,**F**) Number of genes exhibiting parental bias in expression related to *Camellia azalea*-specific (**E**) and *C. amplexicaulis*-specific methylation sites (**F**) in F_1_ hybrids. C.aza-bias, genes showing biased expression toward the alleles from *C. azalea*; C. amp-bias, genes showing biased expression toward the alleles from *C. amplexicaulis*; No-bias, genes showing no parental bias in expression. (**G**,**H**) Venn diagram showing the number of genes co-regulated by *C. azalea*-specific and *C. amplexicaulis*-specific methylation sites within gene groups not exhibitin (**G**) or exhibiting (**H**) parental bias. C.aza-SMS-gene, genes regulated by *C. azalea*-specific methylation sites; C.amp-SMS-gene, genes regulated by *C.* *amplexicaulis*-specific methylation sites. (**I**) Distribution of mean allelic methylation levels of alleles simultaneously influenced by PSMSs. The methylation levels of parental alleles were assessed by calculating the mean methylation rate at PSMSs. Aaza, allele from *C. azalea*; Aamp, allele from *C. amplexicaulis*. (**J**,**K**) Heatmap showing the allelic expression of genes subject to maternally (**J**) and paternally (**K**) monoallelic methylation. Legend shows normalized gene expression levels. Orange, high expression; green, low expression; Aaza, allele from *C. azalea*; Aamp, allele from *C. amplexicaulis*.

By mapping parental transcriptome sequencing reads to the reference genome of *C. sinensis*, parent-specific single-nucleotide polymorphisms (PSNPs) were identified. During these processes, 236,338 PSNPs (located in 6994 genes) were distinguished between *C. azalea* and *C. amplexicaulis*. Gene transcript abundances were then quantified as normalized read counts mapped per SNP site. Trimmed Mean of M-values (TMM) method, implemented in the edgeR package (version 3.38.4), was applied for data normalization across libraries under the assumption that most genes are not differentially expressed (Figure 5A). Based on PSNPs, allelic expression was analyzed. As shown in Figure 5B,C, the expression of paternal and maternal alleles in F_1_ hybrids exhibits a strong linear correlation with the overall expression, which can be quantified as y = −0.95 + 0.99x (*R*^2^ = 0.94) and y = −1.06 + 1.01x (*R*^2^ = 0.95), respectively. Subsequently, by applying the negative binomial distribution and a 1.5-fold differential expression threshold, a total of 1222 pairs of differentially expressed alleles were identified in the F_1_ generation, with 560 showing maternal bias and 662 showing paternal bias in expression (Figure 5D).

### 2.7. Allele-Specific Methylation Patterns in the F_1_ Hybrids

To investigate the effect of DNA methylation, particularly allele-specific methylation (ASM), on AEI, whole-genome resequencing was performed on both parental and F_1_ hybrid generations to comprehensively identify parent-specific methylation sites (PSMSs). In total, 103.62 Gb clean data were obtained after data filtration (Table 3). The sequencing depth was approximately 12× coverage of the genome, with Q20 over 98%. The clean data were then mapped to the genome of *C. sinensis*, and the properly paired mapping rates for the male parent, female parent and F_1_ hybrid were 77.90%, 74.91% and 74.82%, respectively. After sequence alignment and SNP calling, 910,170 and 1,585,161 SNPs were detected in the parental species of *C. azalea* and *C. amplexicaulis*, respectively. Among these SNPs, 231,931 were *C. azalea*-specific C-to-other substitutions, and 425,372 were *C. amplexicaulis*-specific C-to-other substitutions. PSMSs were identified by mutual verification between resequencing and WGBS data. Finally, 122,632 *C. azalea*-specific and 60,350 *C. amplexicaulis*-specific methylation sites were obtained, located in 5510 and 4379 genes with allelic expression data, respectively.

Integrating analysis was conducted to reveal the relationship between PSMSs and AEI. The results showed that most (4571, 82.96%) of the 5510 genes associated with *C. azalea*-specific methylation sites showed no parental bias in expression, while only 418 (7.59%) and 521 (9.46%) exhibited maternal and paternal bias, respectively (Figure 5E). Similarly, for *C. amplexicaulis*-specific methylation sites, 83.32% (3649) of the 4379 associated genes were not biasedly expressed, with 330 (7.54%) and 400 (9.13%) showing maternal and paternal bias, respectively (Figure 5F). Furthermore, over 60% of the alleles were co-regulated by both paternal and maternal allele-specific methylation sites (Figure 5G,H). Specifically, 3238 (64.99%) out of 4982 non-allelically biased expressed alleles were co-regulated by biparental PSMSs (Figure 5G). Among the 1023 genes exhibiting allelic expression bias, 646 (63.15%) demonstrated biparental PSMS regulation (Figure 5H). Notably, only 293 (28.64%) and 84 (8.21%) of these biasedly expressed genes showed exclusive regulation by maternal or paternal PSMSs, respectively (Figure 5H).

We further analyzed the allelic methylation level of genes co-regulated by biparental PSMSs using the *Wilcoxon* test and the *T*-test, sequentially. The methylation levels of parental alleles in F_1_ hybrids were assessed by calculating the mean methylation level at PSMSs. As shown in Figure 5I, the median methylation level of *C. amplexicaulis*-derived alleles was slightly higher than that of *C. azalea*-derived alleles. However, at the individual gene level, nearly all genes exhibited no significant difference in methylation between the two parental alleles. Specifically, all 2433 tested with the *Wilcoxon* test showed no significant difference (*p* < 0.05) in allelic methylation. Similarly, only 2.72% (44) of the 1619 genes tested with the *T*-test exhibited a significant difference (*p* < 0.05) in allelic methylation. We then analyzed the relationship between ASM and its impact on AEI. Among the 293 genes with maternally monoallelic methylation, 127 (43.34%) showed maternal expression dominance, while 166 (56.66%) exhibited paternal bias. Of the 84 genes with paternally monoallelic methylation, 39 (46.43%) showed maternal expression dominance, and 45 (53.57%) exhibited paternal bias (Figure 5J–K).

## 3. Discussion

### 3.1. Cytosine Methylation Remodeling in Interspecific F_1_ Hybrids

Epigenetic changes, as an important response mechanism of organisms to shocks, have become one of the focal concerns in biology. Significant alterations in DNA methylation levels have been detected in many plant hybrids. Liu et al. [19] found, in their study on maize, that the overall DNA methylation levels in all tissues of hybrid offspring were lower than the average mid-parent values (MPVs), and demethylation events were significantly more frequent in hybrids compared to their parents. Similarly, Wang et al. [20] reported in their research on *Chrysanthemum morifolium* and *Leucanthemum paludosum* that the degree of DNA methylation was significantly reduced in the hybrid offspring of these two species. The widely accepted view is that DNA methylation is critical for transcriptionally silencing transposons. So, the relaxation of methylation in hybrids indicates an increased probability of transposon transposition [21,22]. In this study, chromosome-wide analysis revealed that the overall methylation level of the F_1_ hybrids was intermediate between the two parental lines; however, the methylation levels of nearly all chromosomes in hybrids were higher than the mid-parent values (MPVs) in CG, CHG, and CHH contexts (Figure 2J–L). That means F_1_ hybrids did not show a significant decrease in overall methylation compared to the parents. Our previous comparative transcriptome study on the hybridization of *C. azalea* and *C. amplexicaulis* detected a higher level of transcriptional activity of transposable elements (TEs) in the F_1_ hybrids, which indicated that the transcriptional activity of TEs was activated after hybridization [17]. Meanwhile, many genes essential for methylation, especially RNA-directed DNA methylation (RdDM), were also upregulated in the F_1_ hybrids [17]. Considering that most (>60%) of the methylated sites were located in CpG islands and repetitive genomic regions (Figure 2D–F), we believe that methylation indeed plays a crucial role in suppressing TE activity. The relaxation of methylation leads to increased TE mobilization in hybrids. However, de novo methylation in hybrids may elevate overall methylation levels to a certain extent, thereby restricting TE activity to a manageable range, as large-scale transposition could compromise genomic stability. The RdDM pathway likely plays a significant role in this process. This viewpoint can be substantiated by studies on the hybrids of two *Arabidopsis* ecotypes, whose hybrids displayed increased DNA methylation across their entire genomes, and increased methylation of the hybrid genomes predominantly occurred in regions covered by small RNAs [23]. Shen et al. [23] thought that the RdDM pathway may direct DNA methylation in hybrids. Taken together, we suspect that the methylation level returns to a certain level, which may be helpful for maintaining the chromatin and genome stability of the hybrids.

### 3.2. Inheritance Patterns of Cytosine Methylation in Interspecific F_1_ Hybrids

The question of whether methylation patterns can be inherited or transmitted across generations has long been controversial. It is generally believed that methylation patterns can be stably inherited in mitotic somatic cells but undergo extensive erasure during germline development from one generation to the next [24,25]. However, more and more studies have revealed a wide range of special cases of transgenerational epigenetic inheritance [26,27]. Cytosine methylation, as a widespread epigenetic mechanism in plants, has also proved to be heritable across mitotic and meiotic cell divisions [28]. In the pollen of *Arabidopsis*, more than 80% of mC residues were retained, including all those in a symmetric sequence context (CG or CHG, where H is A, C, T) [29]. Similarly, many parental differentially methylated regions (DMRs) at CG and CHG sites were maintained in the rice F_1_ hybrids [14]. In this study, we investigated the transmission and variation patterns of cytosine methylation in the F_1_ hybrids of *Camellia* at both the DMR and DMS levels. At the DMR level, 10.14% of parental CG DMRs were transmitted to the F_1_ generation, exhibiting methylation levels consistent with the paternal parent, compared to 18.48% being consistent with the maternal parent (Figure 3D). A similar pattern was observed in CHG DMRs, with 8.74% of parental CHG DMRs in the F_1_ hybrids showing methylation patterns identical to the mother compared to 5.84% to the father. At the DMS level, over 30% of the DMSs showed parental-biased inheritance pattens (i.e., “Low-parent” and “High-parent”) in the F_1_ hybrids. Although most differential methylation patterns in DMSs could not be clearly defined algorithmically, biased parental inheritance predominated among the non-additive DMSs. These results further indicate that the methylation states of the parental genomes can be stably transmitted to the next generation to a certain extent. However, the inheritance proportions of parental DMRs varied across the three methylation sites. Specifically, DMRs at CG sites appeared more likely to be passed on to the next generation, while those at CHH sites had a much lower probability of being inherited (Figure 3D–F). In fact, plants have developed a set of mechanisms for the maintenance and re-establishment of methylation during evolution. Heritable cytosine methylation primarily occurs in the context of the symmetric CpG dinucleotide, which is maintained by DNA methyltransferase 1 maintenance methyltransferase (MET1) [30], whereas methylation of CHG and CHH sites is maintained by the enzymes CHROMOMETHYLASE 3 (CMT3) and CMT2, respectively [31]. In contrast, the re-establishment of all sequence contexts (CG, CHG, and CHH) is guided by small RNA or heterochromatin-directed methylation pathways. Different maintenance mechanisms may lead to distinct patterns of methylation inheritance, which could explain the varying inheritance proportions of parental DMRs across the three methylation sites. Heritable methylation variants may contribute to the phenotypic formation of F_1_ hybrids. Therefore, we observed that a series of genes related to plant development were enriched in the DMRs. Of course, many regions without parental DMRs exhibited extensive methylation remodeling, which was consistent with the results reported by Ma et al. [14]. The remodeling of parental DNA methylation is also a widespread phenomenon during hybridization and is of significant biological significance, warranting further investigation in the future.

### 3.3. Cytosine Methylation Balances Parental Allele Expression in Interspecific F_1_ Hybrids

As described above, AEI plays a central role in generating novel hybrid phenotypes. Cytosine methylation is a critical epigenetic regulatory mechanism, and its genomic localization determines its potential to exhibit allele-specific effects and contributes significantly to AEI. However, the extent to which cytosine methylation influences allelic expression remains unclear. In this study, we found that the vast majority of PSMSs associated with allele pairs showed no significant expression bias in the F_1_ hybrids. Meanwhile, we also found that over 60% of non-biasedly expressed alleles were simultaneously influenced by both paternal and maternal allele-specific methylation sites (Figure 5G–H), and the proportion of genes subject to monoallelic methylation is actually very small in F_1_ hybrids. This suggests allele-specific methylation (ASM) is not prevalent in F_1_ hybrids. One important reason for this is that biparental PSMSs are widespread and often act on the same allele pairs. These PSMSs may recruit distinct chromatin-modifying enzymes (such as DNA methyltransferases and demethylases), thereby creating a counteracting modification environment in F_1_ hybrids, resulting in no significant bias in the methylation levels between the two alleles. Consequently, although the methylation state of over 30% of the DMSs could be parental-biasedly transmitted to the F_1_ generation, the vast majority of alleles do not exhibit biased expression. In this sense, PSMSs play a crucial role in balancing the allelic expression in F_1_ hybrids. However, when it comes to F_2_ and subsequent generations or backcross populations, the composition and distribution of species-specific sites may change due to genomic recombination, thereby rewriting the methylation patterns of alleles and their expression.

Our research can also provide valuable insights into the study of the relationship between genetics and epigenetics. Previous studies have demonstrated that allele-specific DNA methylation patterns are sequence-dependent [12,13,32]. Our findings suggest that methylation divergence between parental alleles in F_1_ hybrids is largely governed by the stochastic distribution of PSMSs. The random nature of mutation sites between parents leads to an even distribution of the PSMSs across allele pairs in F_1_ hybrids, thereby minimizing methylation divergence between the parental alleles. These results support the epigenetic neutral hypothesis, which posits that most methylation variations are passively inherited from genetic polymorphisms [33]. Building on this, if the parental genomes are available, the methylation patterns of alleles in hybrid offspring should be partly predictable, especially in the era of big data and artificial intelligence (AI). In fact, similar studies and reports have already emerged [34]. Future studies should account for the stochastic nature of cytosine site distribution when interpreting methylation divergence in hybrids.

### 3.4. Multiple Allele-Specific Sites May Critically Shape Phenotypes of Interspecific F_1_ Hybrids

Although allele-specific methylation is not prevalent in F_1_ hybrids, we still detected 293 and 84 genes subject to maternally and paternally monoallelic methylation, respectively. Among the 293 genes with maternally monoallelic methylation, 166 (56.66%) exhibited paternal expression dominance. These genes included F-box protein coding gene *At5g07610*, *DEFA*, NAC domain-containing protein coding gene, *bHLH147* and other genes. *At1g13570* belong to the F-box protein family, and they have been implicated in regulating floral organ development [35]. The *DEFA* gene belongs to the B-class MADS-box gene family and can regulate plant floral development through multiple pathways [36]. The NAC transcription factors also play core roles in flowering development [37]. Of the 84 genes with paternally monoallelic methylation, 39 (46.43%), including F-box/WD-40 repeat-containing protein coding gene *At5g21040*, *factor of DNA methylation* (*FDM2*), showed maternal expression bias (Figure 5). *FDM2* is required for the RNA-directed DNA methylation pathway [38] and is highly expressed in the shoot apex during the transition from the vegetative to the inflorescence stage and in flowers at early developmental stages [39]. Based on the above results, we speculate that allele-specific methylation continues to play an important role in shaping allelic expression and phenotypes of F_1_ hybrids, although this role is dependent on sequence variation and accompanied by stochasticity. In addition, we also found that in a significant proportion of alleles, ASM did not exhibit the corresponding negative regulatory relationship with allelic expression and even showed the opposite pattern. We speculate that these alleles may be simultaneously influenced by other regulatory factors. Previous studies showed that miRNAs [40], SiRNA [41], and transposable elements [42] could also act on a single allele to cause AEI. Partial monoallelic methylation does not always lead to AEI, which could be due to the interaction of these factors that balance the expression of the two alleles. This phenomenon warrants further investigation in future studies.

## 4. Materials and Methods

### 4.1. Plant Materials

In this study, two diploid species of *Camellia azalea*, *C. amplexicaulis* as well as their F_1_ offsprings were used to study the cytosine methylation change after hybridization (Figure 1B). Hybridizations were conducted by Palm Eco-Town Development Co., Ltd. following the technical process described by Gao et al. [43], taking *C. azalea* as the female parent and *C. amplexicaulis* as the male parent. Specifically, pollens from different individuals of *C. amplexicaulis* were collected together; the mixed pollens were then used to pollinate the flowers of *C. azalea* plants. So, the F_1_ hybrids may be not from identical parents, but their parents came from individuals of one wild population. All the plants used in this study were grown in the same greenhouse of the Palm Eco-Town Development Co., Ltd. (Gaoyao, China) under a natural light condition. Voucher specimens of these collections were preserved in the Herbarium of Nanjing Forestry University (NF) with accession numbers of Caza703, Camp706 and Caza_Camp707. Flower buds of the F_1_ hybrids and the parental species at the same stage were harvested and frozen in liquid nitrogen immediately, then transferred to −80 °C refrigerator for storage.

### 4.2. Whole-Genome Bisulfite Sequencing

Whole-genome bisulfite sequencing was conducted following a standard workflow. The general process is as follows: Total DNA was extracted from flower buds using the Super Plant Genomic DNA Kit (DP360, TIANEN, Beijing, China) according to the manufacturer’s instructions. For the parental species and F_1_ hybrid, three biological replicates (from three randomly selected individuals) were set up. Three DNA pools were constructed by mixing equal quantities of DNA from the three individuals, respectively. The mixed DNA was then fragmented by sonication using a Bioruptor (Diagenode, Liège, Belgium) to a mean size of approximately 250 bp, followed by blunt-ending, dA addition to the 3′-end, and adaptor ligation, essentially according to the manufacturer’s instructions. Subsequently, the ligated DNA was bisulfite-converted using the EZ DNA Methylation-Gold Kit (Zymo, Irvine, CA, USA). Fragments of different insert sizes were excised from the same lane of a 2% TAE agarose gel. The products were purified using the QIAquick Gel Extraction Kit (Qiagen, Valencia, CA, USA) and amplified by PCR. Finally, the products were sent to Beijing Genomics Institute (BGI, Shenzhen, China) for high-throughput sequencing.

### 4.3. Data Filtering and Sequence Alignment

The raw data were filtered with an in-house Perl script. Clean data were obtained by removing reads with adapters, ambiguous reads with more than 10% of unknown bases, and low-quality reads with over 10% of low quality (quality value < 20). After filtering, the remaining reads were mapped to the genome of *C. sinensis* [18] using the BSMAP software (version 2.74) [44]. Mapping rate and bisulfite conversion rate of each sample were then calculated.

### 4.4. Differentially Methylated Regions Detection

Methylation level was evaluated as the mC/C ratio at each reference cytosine site. Differentially methylated regions (DMRs) were identified by comparing methylation levels in genomic windows (with ≥5 CG/CHG/CHH sites) between two samples, and regions showing ≥ 2-fold methylation differences (Fisher test *p* ≤ 0.05) were defined as DMRs.

### 4.5. Differentially Methylated Site Detection and Inheritance Pattern Classification

Fisher’s exact test was used to identify the pairwise differentially methylated sites (DMSs) between F_1_ hybrid and its parents. *p*-values were calculated based on the hypergeometric distribution, and a threshold of *p* < 0.01 was applied to identify significant DMSs. The pairwise DMSs were further categorized into eight groups (Figure 4A,B), as described by Chen et al. [45]. Specifically, DMSs with methylation levels intermediate between the two parents were classified as “Additive”. Non-additively methylated DMSs were subdivided into four groups when the parents exhibited different methylation levels: DMSs with methylation levels in the hybrid similar to those of the low-parental or high-parental value were classified as “Low-parent” or “High-parent”, respectively; DMSs with methylation levels in the hybrid higher than the high-parental or lower than the low-parental value were defined as “Above high-parent” and “Below low-parent”. When the methylation levels of the parents were equal, non-additively methylated DMSs were further classified into two groups: DMSs with methylation levels in the hybrid higher or lower than both parents were defined as “Above parent” or “Below parent”. Finally, DMSs that could not be clearly defined algorithmically were classified as “Ambiguous”.

### 4.6. Transcriptome Data Analysis

The transcriptome data were generated from our previous study [46]. We conducted a re-analysis of the data by focusing on allelic expression in F_1_ hybrids. The materials used for RNA-sequencing were the same batch as those used for methylation sequencing in this study. After data filtering, the clean data were mapped to the genome of *C. sinensis* [18] using STAR software (version 020201) [47]. SNP calling, species-specific SNP identification was conducted following the methods described in our previous study [46]. Trimmed Mean of M-values (TMM) strategy, implemented in the edgeR package [48], was applied for data normalization across libraries under the assumption that most genes are not differentially expressed. Based on species-specific SNPs, allelic gene expression was analyzed. Differentially expressed alleles were identified by applying the negative binomial distribution and a 1.5-fold differential expression threshold.

### 4.7. Whole-Genome Resequencing and Parent-Specific Methylation Sites Identification

The three DNA pools, as described above, were also used for whole-genome resequencing. In total, three libraries were constructed and then paired-end sequenced using the BGISEQ platform (BGI, Shenzhen, China) with the standard resequencing protocols. FastQC (https://www.bioinformatics.babraham.ac.uk/projects/fastqc/ (accessed on 17 July 2025)) was used for data filtering. After filtering, clean reads were mapped to the reference genome of *C. sinensis* [18] using the BWA software (version 0.7.17-r1188) [49]. Then, SAMtools (version 1.9) [50] and VarScan (version 2.3.9) [51] software were orderly used for SNP calling. Parent-specific methylation sites (PSMSs) were identified by integrating the resequencing data and WGBS data following the principles as follows: (i) the SNP sites in the two parents must be homozygous for difference; (ii) each SNP site in the F_1_ hybrid must consist of only two alleles (one for the male parent, another for the female parent); (iii) the SNP calls from resequencing must be consistent with those from WGBS; (iv) the number of reads supporting methylation at non-cytosine sites must be zero; (v) cytosine sites in F_1_ hybrids must be methylated.

## 5. Conclusions

In this study, we employed multi-omics analysis to investigate the inheritance of DNA methylation patterns and their regulatory role in allelic expression in F_1_ hybrids. The results revealed that the whole-genome methylation patterns underwent remodeling following hybridization. Parental-biased inheritance predominated among the non-additive differentially methylated sites (DMSs), and the methylation state of ~30% of the DMSs could be parental-biasedly transmitted to the F_1_ hybrids. Allele-specific methylation (ASM) was not universally observed, primarily due to the random distribution of parent-specific methylation sites (PSMSs). Approximately 60% of the non-biasedly expressed alleles were simultaneously influenced by both paternal and maternal allele-specific methylation sites. We propose that, rather than causing allelic expression imbalance, the randomly distributed PSMSs play a more significant role in balancing allelic expression in F_1_ hybrids. Consequently, most alleles in F_1_ hybrids exhibited unbiased expression. However, this does not mean that ASM is unimportant in F_1_ hybrids; on the contrary, once it occurs, it may hold significant biological implications in modulating AEI and transgressive phenotype in the F_1_ hybrids. These results reflect the synergistic role of genetic and epigenetic factors in shaping allelic expression and phenotypes. It is worth noting that our study focused specifically on ASM and its regulatory patterns in F_1_ hybrids. It can be envisioned that in F_2_ and subsequent generations, as well as in backcross populations, the patterns of allele methylation and their regulatory mechanisms will undergo substantial changes due to genomic recombination and alterations in genetic composition. These aspects warrant further investigation.

## Figures and Tables

**Table 1:** Summary of whole-genome bisulfite sequencing and alignment.

Sample ID	Clean Data Size (bp)	Mapped Reads	Mapping Rate (%)	Uniquely Mapped Reads	Uniquely Mapping Rate (%)	Bisulfite Conversion Rate (%) *	Average Depth (X)
C.amp	103,604,864,200	771,474,941	74.46	566,814,667	54.71	99.39	16.57
F_1_	108,717,190,200	761,566,451	75.44	548,873,456	54.37	99.38	17.12
C.aza	109,177,865,800	809,930,929	74.18	578,004,884	52.94	99.34	16.94

Note: * Bisulfite Conversion Rate = 1—methylation rate of Lambda DNA. C.aza, *Camellia azalea*; C.amp, *C. amplexicaulis*; F_1_, F_1_ hybrids.

**Table 2:** The number of methylated cytosines (mCs) in the F_1_ hybrids and their parents.

Sample ID	mC Number			
	mCG	mCHG	mCHH	Total
C.amp	35,035,237	39,065,979	93,074,792	167,176,008
F_1_ hybrid	43,862,913	49,018,324	123,429,484	216,310,721
C.aza	42,652,315	47,769,161	119,565,996	209,987,472

Note: C.aza, *Camellia azalea*; C.amp, *C. amplexicaulis*; F_1_, F_1_ hybrids.

**Table 3:** Summary of whole-genome resequencing.

Sample ID	Clean Reads	Clean Base	Read Length	Q20 (%)	GC (%)
C.amp	120,130,447	36,039,134,100	PE150	98.53	37.77
F_1_	107,593,530	32,278,059,000	PE150	98.29	39.02
C.aza	117,701,194	35,310,358,200	PE150	98.37	39.15

Note: C.aza, *Camellia azalea*; C.amp, *C. amplexicaulis*; F_1_, F_1_ hybrids.

## Data Availability

All the sequencing data are available at the NCBI Sequence Read Archive (SRA) database under the BioProject of PRJNA1362437.
